# Magnetically sensitive nanodiamond-doped tellurite glass fibers

**DOI:** 10.1038/s41598-018-19400-3

**Published:** 2018-01-19

**Authors:** Yinlan Ruan, David A. Simpson, Jan Jeske, Heike Ebendorff-Heidepriem, Desmond W. M. Lau, Hong Ji, Brett C. Johnson, Takeshi Ohshima, Shahraam Afshar V., Lloyd Hollenberg, Andrew D. Greentree, Tanya M. Monro, Brant C. Gibson

**Affiliations:** 1 ARC Centre of Excellence for Nanoscale BioPhotonics, Institute of Photonics and Advanced Sensing, The University of Adelaide, Adelaide, SA 5005 Australia; 2 School of Physics, University of Melbourne, Parkville, VIC 3010 Australia; 3 Chemical and Quantum Physics, School of Science, RMIT University, Melbourne, VIC 3001 Australia; 4 ARC Centre of Excellence for Nanoscale BioPhotonics, School of Science, RMIT University, Melbourne, VIC 3001 Australia; 5 Centre for Quantum Computing and Communication Technology, School of Physics, University of Melbourne, Parkville, VIC 3010 Australia; 6 National Institutes for Quantum and Radiological Science and Technology (QST), Takasaki, Gunma 370-1292 Japan; 7 University of South Australia, Adelaide, SA 5000 Australia

## Abstract

Traditional optical fibers are insensitive to magnetic fields, however many applications would benefit from fiber-based magnetometry devices. In this work, we demonstrate a magnetically sensitive optical fiber by doping nanodiamonds containing nitrogen vacancy centers into tellurite glass fibers. The fabrication process provides a robust and isolated sensing platform as the magnetic sensors are fixed in the tellurite glass matrix. Using optically detected magnetic resonance from the doped nanodiamonds, we demonstrate detection of local magnetic fields via side excitation and longitudinal collection. This is a first step towards intrinsically magneto-sensitive fiber devices with future applications in medical magneto-endoscopy and remote mineral exploration sensing.

## Introduction

The sensing of magnetic fields is important for applications as diverse as mining exploration^1^ and aircraft navigation^2^. Within medical fields, applications such as magneto-encephalography^3^ and magneto-cardiology^4^ are important methodologies for sensing spatially-resolved activity in the brain and heart, respectively. Although existing magnetometers such as superconducting quantum interference devices (SQUIDs) and optical atomic magnetometers are ultrasensitive to magnetic fields^5^, they are constrained in terms of their size, cost and temperature of operation. Optical fibers provide an ambient, robust, cheap and alternate approach for remote magnetic sensing. Optical fiber Bragg gratings have shown sensitivity to magnetic fields with thick coatings of magnetostrictive materials such as Terfenol-D applied in a polymeric matrix^6,7^. Multimode interference effects in spliced square no-core optical fiber surrounded by magnetic fluid also exhibit magnetic sensitivity^8,9^. However, for both of these solutions, the magnetic sensitive materials are external to the optical fibers. Solid state magnetometers based on the negatively-charged nitrogen-vacancy (NV) defect in diamond provide an additional avenue for sensitive magnetic field detection under ambient conditions^5,10^. The size of such systems can be of order nanometers opening up new opportunities for robust, miniature and remote magnetic sensors. Recent work has demonstrated magnetic sensitivities to oscillating fields of $\sim 1\,\mathrm{pT}/\sqrt{{\rm{Hz}}}$ from sensing volumes of order 100 µm^2^ in bulk single crystal diamond^11^ and $\sim 290\,\mathrm{nT}/\sqrt{{\rm{Hz}}}$ for nanodiamond (ND) crystals^12^.

The magnetic field sensitivity for NV-based systems is dependent on the fluorescence collection efficiency. Conventional detection approaches utilize high numerical aperture objectives to collect isotropic emission from diamond defect centers. This approach yields a typical collection efficiency of emitted photons around 2%^13^ but is limited by the size of the objective and mechanical instabilities. One attractive solution to this problem is to couple the NV fluorescence emission into guided modes of a waveguide, thereby reducing the overall size of the collection optics and enabling integration with mature photonic technologies. Several approaches have been investigated toward this goal, with diamonds grown on the endface of fibers^14,15^, and NDs manipulated on to the end face^16^, or tapered regions^17^. The spin properties of the NV centers have been explored in these geometries^16,18^, however the sensor remains external to the glass leading to a lack of mechanical stability, limiting the device’s applicability and lifetime. To overcome these limitations, we explore a hybrid strategy with ND-doped within a tellurite glass matrix^19,20^. Previously, NV centers in NDs embedded in tellurite glass have shown single-photon emission^21^, as well as efficient coupling to tellurite microspheres^22^.

Here we show the magnetic response from tellurite doped fibers. The doped ND fibers are excited transverse to the optical fiber axis with the NV emission coupled into the guided modes of the tellurite glass fibers. Using optically detected magnetic resonance from the NDs we demonstrate the intrinsic sensitivity of the doped tellurite fiber to external magnetic fields.

## Results and Discussions

### NV emission coupled to the tellurite fiber

**Figure 1:**
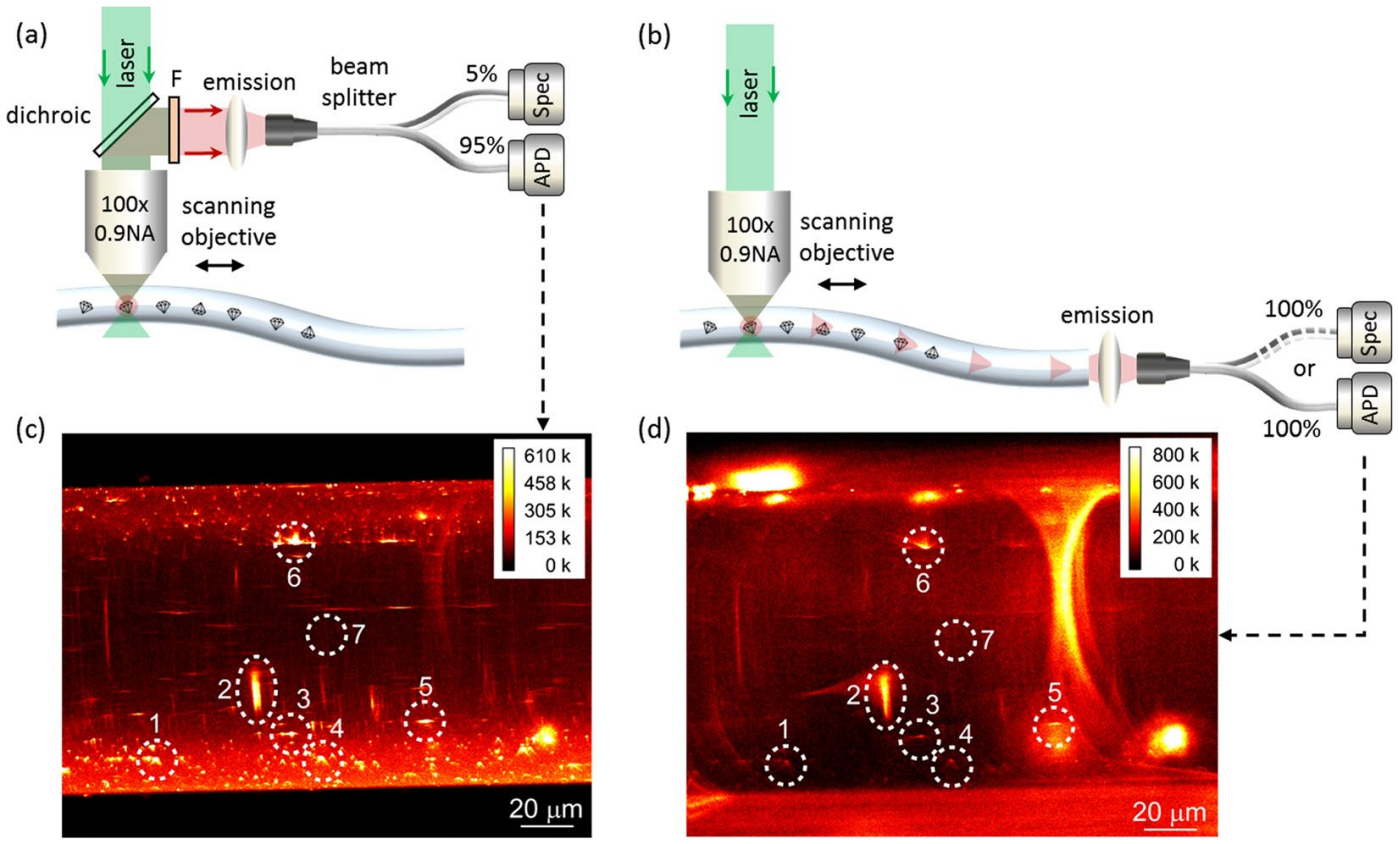
(**a**) and (**b**) Schematic of side pumping setup for fluorescence mapping based on a custom scanning confocal microscope. In (**a**), the fluorescence signal is collected from the side with a high NA objective and split into 5% and 95% for spectral and intensity measurements, respectively. In (**b**), the fluorescence signal is collected from the ND-doped fiber output endface using a multimode fiber that is manually connected to the spectrometer or APD for spectral and intensity measurements, respectively. F: 560 nm long-pass filter, Spec: spectrometer, APD: avalanche photodiode detector. (**c**) and (**d**) are images of the scanned ND-doped fiber plane parallel to the fiber axis. (**c**) was collected using the side-collection setup shown in (**a**), and (**d**) was collected using the endface-collection setup shown in (**b**). The same seven regions are numbered in (**c**) and (**d**), showing these points could be spatially recovered through the fiber.

Characterisation of the ND-doped tellurite fiber was performed in two separate geometries, the first involved confocal imaging transverse to the fiber as shown in Fig. 1a. The second used transverse excitation and remote endface collection, see Fig. 1b. A typical fluorescence image using the confocal imaging geometry reveals isolated emission from doped NDs, as shown in Fig. 1c. The excitation spot in this geometry is distorted in the axial direction due to the cylindrical shape of the fiber. An analogous image can be acquired by scanning the transverse excitation beam and collecting the fluorescence from the fiber endface. This resulting fluorescence image from this geometry is shown in Fig. 1d. The endface image reproduces the isolated fluorescence signals from the doped NDs. This demonstrates coupling of the ND emission to the guided modes of the fiber.

**Figure 2:**
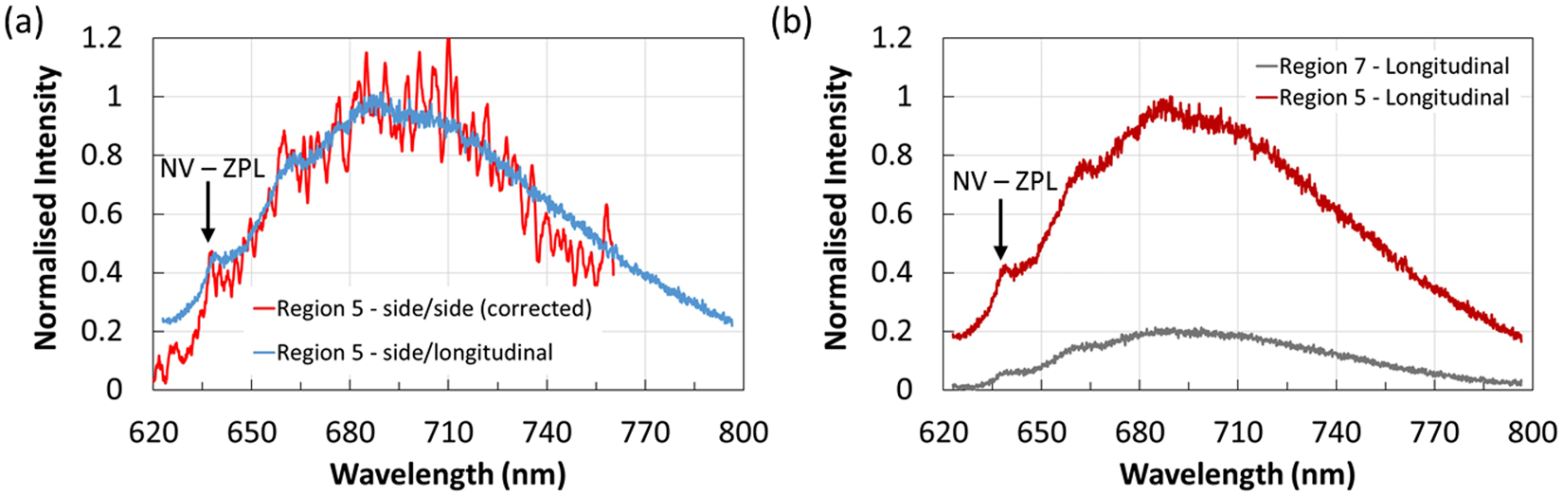
Fluorescence spectrum from ND doped tellurite fibers. (**a**) Comparison of the fluorescence spectra obtain from the side excitation and side (blue) and longitudinal (red) collection geometries. The NV zero phonon line (ZPL) is marked at 637 nm. (**b**) Spatially resolved fluorescence spectra excited from two distinct locations Region 5 and 7 from Fig. 1d and collected in the longitudinal geometry from the fiber endface.

To verify NV emission, fluorescence spectra were measured under side excitation and collection, Fig. 1(a), and side excitation and longitudinal collection as shown in Fig. 1(b). In both cases, the spectra show characteristic NV emission with a zero-phonon-line (ZPL) at 637 nm, see Fig. 2(a). The fluorescence spectra were acquired under the same exposure time and when intensity corrected to account for the 95/5 ratio of the fibre coupler in Fig. 1(a) both geometries report similar NV fluorescence intensities. An interesting point in comparing the two collection geometries under side excitation is that the endface image is able to reproduce the spatial distribution of NDs. An example is shown in Fig. 2b with the spectra obtained from the fiber endface with the excitation laser focused on separate spatial locations. The NV emission when exciting Region 5 (see Fig. 1d) of the tellurite fiber is a factor of 5 times greater than the background from Region 7. This opens up the possibility to report multiple NDs which are spatially separated within the fiber.

### ODMR characterization of the ND-doped tellurite fiber

**Figure 3:**
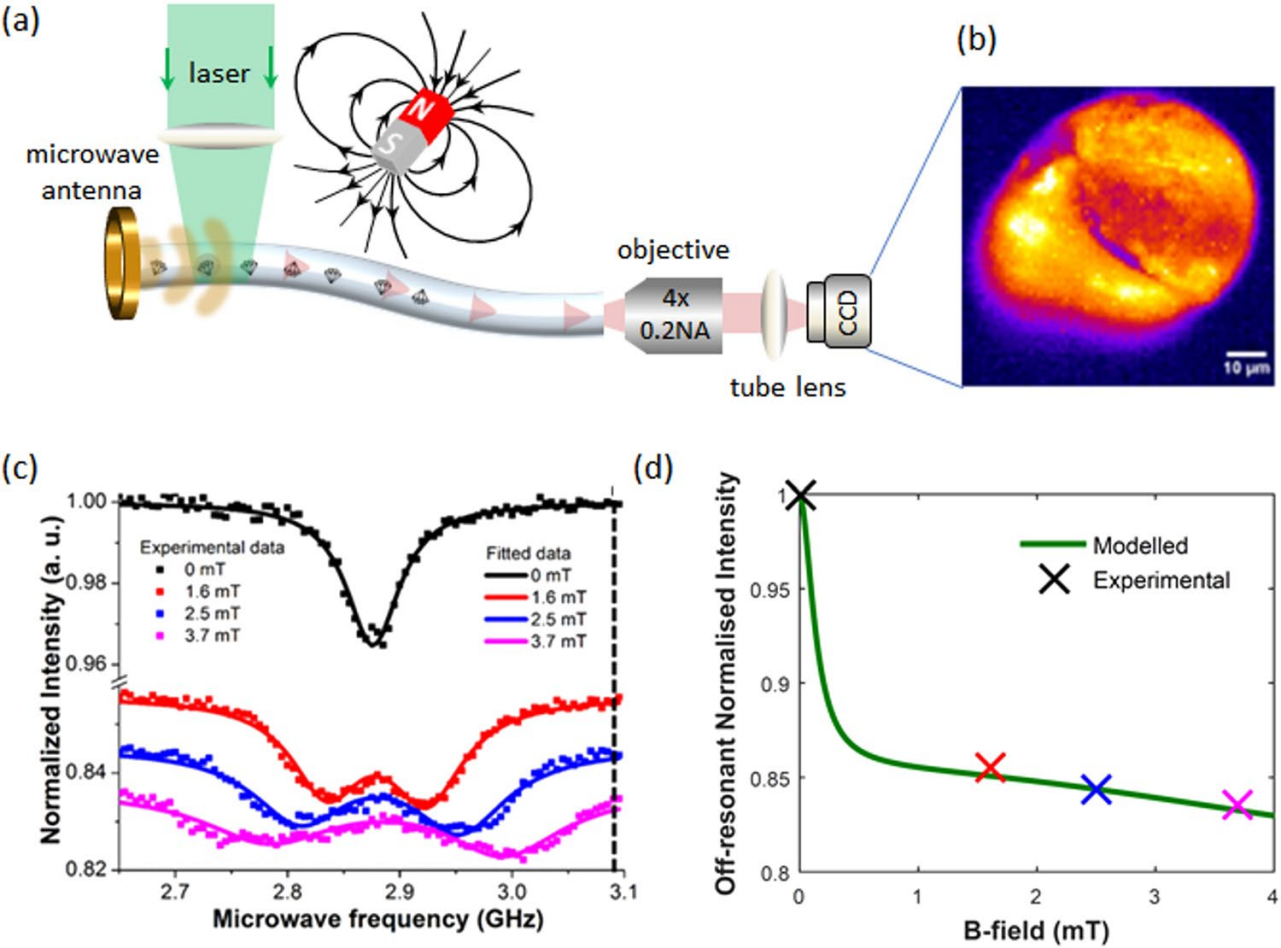
Optically detected magnetic resonance of ND doped tellurite fiber. (**a**) Experimental schematic for ODMR in the tellurite fiber. The fiber was excited from the side with green laser light at 532 nm, approximately 5 cm away from the fiber endface. The NV emission from the endface was imaged onto an sCMOS camera, as shown in (**b**). (**c**) ODMR spectra from a ∼200 µm^2^ region of the fiber for varying external magnetic fields. The spectra were obtained by integrating the fluorescence signal from the full field of view. The square points are measured data, and the solid lines are fitted curves. (**d**) Theoretical model of the off-resonant fluorescence intensity versus magnetic field. Crosses are the data extracted from (**c**) at 3.1 GHz.

To determine the magnetic response of the tellurite doped fiber, we performed optically detected magnetic resonance (ODMR) on NDs in the presence of an external magnetic field as shown in Fig. 3a. The doped fiber was optically pumped from the side and the NV fluorescence collected and imaged from the endface of the fiber, see Methods. The ground state energy levels of the NV centre are paramagnetic spin states and can be readout optically due to the fluorescence difference between the m_s_ = 0 and m_s_ = ±1 states. Under CW optical excitation, the electronic spin of the NV centre is conveniently polarized into the m_s_ = 0 state. The presence of an external magnetic field Zeeman splits the m_s_ = ±1 states with a gyromagnetic ratio 28 GHz/T. This shift can be recorded optically with the addition of microwave excitation to drive the NV spin from the bright m_s_ = 0 to the darker m_s_ = ±1 state. Off-axis magnetic fields can result in spin mixing, this has also been used to detect external magnetic fields, all optically^23^.

Figure 3b shows a typical fluorescence image from the fiber endface. The inhomogeneous distribution from the cross-section of the fiber confirms that the emission originates from a collection of spatially distributed incoherent sources, i.e. the NVs in the doped NDs. While this relates back to spatial information, the spatial resolution here is less than that achieved with scanning a spatially focused excitation spot in Fig. 1. For the following magnetic field measurements, we therefore integrated the entire endface signal. The ODMR from excitation of a ∼200 µm^2^ focused region of the fiber is shown in Fig. 3c for various external magnetic fields. The square points show the raw ODMR data, while the solid lines show the single or double Lorentzian fits to the measured data points. The intensity signal is normalized to the fluorescence intensity away from resonance at zero magnetic field, conserving the relative intensities between each trace. The black solid curve indicates a zero-field ODMR with a single degenerate dip at 2.876 GHz as expected. This central ODMR resonance was split by the magnetic field as shown as red, blue and violet traces. The ODMR contrast in the presence of an external magnetic field reduces from 10% to ∼3.5% consistent with that observed from NV ensembles in bulk diamond crystals^24^. The ODMR linewidth at zero magnetic field, 28.8 ± 0.8 MHz, is significantly broader than that observed from the NVs ensembles. This is likely caused by several NDs which were probed within the excitation volume and possibly increased strain due to doping inside the fiber, leading to inhomogeneous broadening of the ODMR line. The sensitivity of the magnetometer to DC magnetic fields using Zeeman splitting is determined by the linewidth, contrast and fluorescence count rate^25^:

$${\eta }_{dc}=\frac{4h\delta }{3\sqrt{3\cdot }{g}_{NV}{\mu }_{B}R\sqrt{N}}$$

Where δ = 28.8 MHz is the full width at half maximum of the ODMR peak, R = 0.035 is the ODMR contrast, *g*_*NV*_ = 2.0028 is the g-factor of the NV centre^26^ and µ_B_ is the Bohr magneton. The number of photons per second detected at the output end of the fiber is N = 4×10^6^ s^−1^, resulting in a projected magnetic sensitivity of $\sim 11\,\mu {\rm{T}}/\sqrt{{\rm{Hz}}}$.

In addition to the Zeeman splitting, we also observe an overall reduction in the fluorescence intensity with increasing magnetic field which can be best seen away from the resonance peaks (e.g. at 3.1 GHz). To investigate this fluorescence intensity reduction, we theoretically modelled the impact of the external magnetic field on the fluorescence rate and ODMR signal for varying magnetic field strengths, see Supplementary Material. Using the theoretical model, the off-resonant reduction of the fluorescence signal was reproduced. Figure 3(d) shows how the off-resonant fluorescence intensity at 3.1 GHz changes with magnetic field in our simulation. A quantitative agreement between the theory and experimental data is found by including an additional background fluorescence term which remains constant with magnetic field and has a strength of 37% of the strongest signal (B = 0 mT). Such background fluorescence could stem from auto-fluorescence of the tellurite glass and/or constant ND fluorescence from misaligned NV centers. The off-resonant reduction in fluorescence is due to the fact that spins not aligned with the external magnetic field experience Zeeman-terms perpendicular to the local quantisation axis defined by the NV orientation. These off-axis Zeeman terms generate state mixing between the ‘bright’ m_s_ = 0 and ‘darker’ m_s_ = ±1 spin states. The total fluorescence signal therefore contains contributions from both the off-resonant contributions and the Rabi driving of centers aligned with the magnetic field (conventional ODMR signal). These results are in agreement with previous observations from other researchers^23,27,28^

## Conclusion

Our results demonstrate that diamond-glass hybrid materials have potential to realize a new generation of sensing platforms. In particular, our ND/tellurite glass has been used to realize an inherently magnetic field sensitive optical fiber with sensitivity of order $10\,\mu {\rm{T}}/\sqrt{{\rm{Hz}}}$. Such sensitivity is comparable to that achieved in fiber taper platforms with NDs coated on the taper surface^29,30^. Using a theoretical model that qualitatively reproduces the experimental ODMR results, we have shown that the fiber background fluorescence has a significant impact on intensity and contrast of the ODMR peaks and hence on magnetic sensitivity. Since the entire length of the fiber is doped with NDs, i.e. with nanoscale magnetic sensors, locally pumping a small area of the fiber at different positions along the fiber length offers an additional opportunity to create magnetic gradiometers with enhanced stability and utility.

The effect of a non-aligned magnetic field is to induce spin flips in the NV centers, thereby reducing fluorescence with increasing magnetic field strength, which is in agreement with previous observation^30^. This effect offers all-optical detection of external magnetic fields in the absence of RF fields with our ND-doped fiber approach. Recent work of NV-based magnetometry has shown that all-optical detection greatly reduces the complexity of a practical remote NV magnetometer^23,31^. We also note that the excitation beam intensity was in the range of ∼ 600 W/cm^2^, which could be readily replaced by a green LED for reduced cost and easy alignment.

Optimisation of the magnetic field sensitivity of our ND-doped tellurite fibre is a nontrivial process that requires the understanding of several, competing factors. Certainly more NV centres must lead to more signal, with the final sensitivity ultimately scaling with the square root of the number of optically active NV within the excitation volume, as well as the number of detected photons. To collect more photons, one seeks to minimize optical losses. Our earlier work [19, 20] highlights the role of glass manufacture and purity of the starting materials. However it is also important to stress that Mie scattering from the ND is an intrinsic loss mechanism. To mitigate the effects of Mie scattering suggests that smaller ND centres should be used. However smaller particles are problematic due to oxidation, including complete oxidation, during the melt process. At the same time, decreasing the ND size also leads to a reduction in the NV concentration as NV are typically not found in the proximity of the surface, and hence on surface area to volume ration arguments we would expect the overall NV concentration to reduce with smaller NDs compared with larger NDs. With this in mind, a detailed study into the optimal size of NDs to be incorporated into the glass is important. Surface coatings may also serve to increase the survivability of the NDs, and we note that nickel coatings have been used in the past^32^, although it is not obvious that metallic coatings will be compatible with optical emission from the NVs.

Finally, we note that although tellurite has proven to be a convenient host glass for NDs due to the combination of a low melting point, good transparency, mid-range refractive index, there may well be superior glasses for such inherently sensitive fibers. The search for such combinations is likely to be extremely fruitful.

## Methods

### NV centers in diamond

The NV defect in diamond has a paramagnetic spin-triplet ground state, which can be optically addressed and readout at room temperature^33^. The magnetic sub-levels (m_s_ = ±1) are degenerate at zero magnetic field and separated from the m_s_ = 0 state by the diamond crystal field splitting of D = 2.87 GHz. External magnetic fields Zeeman split the m_s_ = ±1 levels with a gyromagnetic ratio of 28 GHz/T^34^. The position of the magnetic sublevels can be determined optically based on the fluorescence difference between the m_s_ = 0 and m_s_ ±1 states giving rise to optically detected magnetic resonance (ODMR)^35^. The crystal symmetry of the defect results in four possible NV orientations within a single ND. Averaging over a number of NDs in a given volume broadens the ODMR lines and reduces the overall fluorescence contrast, however in practice the contrast remains sufficient to determine the ODMR peak position as shown in Fig. 3.

The NDs used to dope the tellurite glass were manufactured by a non-detonation process with a purity >99.95% (NaBond) and average particle size of 40–50 nm. To create a high number of NV centers, the NDs were irradiated with 2 MeV electrons at a dose of 10^18^ cm^−2^ and then annealed under vacuum at 800 °C for 2 h to mobilise the vacancies to the intrinsic nitrogen, and then in air at 475 °C for 2 h to remove the outer graphitic layer caused by the manufacturing process and irradiation. The resulting NDs contain between 5–15 NV centers per ND.

### NV emission coupled to the tellurite fiber

The ND-doped tellurite optical fiber used for these experiments was the fiber designated as E2 in ref.^20^. The fiber was made in a three step process. First, an ND-doped billet was made using the melt-quench technique, then this billet was extruded into a rod, and finally this rod was drawn down to the fibre. The ND doped tellurite glass billet was fabricated using a two temperature melting procedure. The batch of crystalline raw materials without NDs was melted at a temperature of 690 °C in a silica crucible to obtain a homogenized melt. Then the temperature was reduced to 610 °C, and the ND powder added to achieve a concentration of 12 ppm in weight, and mixed thoroughly into the glass melt. The ND-doped melt dwelled for ~10 min at the second temperature to allow dispersion of the NDs in the melt. Finally, the ND-doped melt was cast into a mold, resulting in the glass billet used for extruding the rod and drawing finally the fibre.

The ND-doped tellurite optical fiber used here has an outer diameter of 160 µm and an optical loss between 9–14 dB/m along the wavelength range from 500 nm to 800 nm^19,20^. The ND concentration in the final fiber was determined as approximately 0.7 ppm in weight^20^, which indicates a significant reduction in diamond material. The lower ND concentration in the fiber compared to the concentration doped into the glass melt is likely caused by oxidation of ND particles by dissolved oxygen in the glass melt during the dwelling time at 610 °C for ~10 min^19^. The ND-doped fibers were excited using a 532 nm continuous laser.

To minimize background fluorescence from impurities in the glass as well as selectively excite NDs in the fiber, we used excitation from the side and custom scanning confocal microscope as shown in Fig. 1a,b. An area close to a fiber endface was scanned in the plane parallel to the fiber axis using a 532 nm diode laser with 7 mW power via a 100× objective (NA 0.9) attached to a nano-positioning stage. The scanned area (137 µm × 200 µm) is on the plane with ∼38 µm vertical physical distance to the fiber surface on the top (close to the objective). Because of the curvature of the fiber surface, the focal plane corresponding to this scanned area was not a fixed distance to the objective. The fluorescence signal from the scanned area was either collected from the side of the ND-doped fiber using the same high NA objective used for excitation (side-collection setup) (Fig. 1a) or from the output endface of the ND-doped fiber using a MM fiber (endface-collection setup) (Fig. 1b). The fluorescence spectra were filtered with a long-pass filter to remove the excitation light.

### ODMR characterization of the ND-doped tellurite fibers

To investigate the magnetic response of the ND-doped tellurite fiber we performed ODMR. The NDs were optically excited via side pumping ∼5 cm from the fiber endface. The 532 nm light was focused with a plano convex lens f = 200 mm (power density of 600 W/cm^2^). The NV fluorescence was collected from the output endface, filtered using two bandpass pass filters 650–750 nm, and then imaged onto a sCMOS camera as shown in Fig. 3. A 2 mm microwave antenna was placed over the ND-doped tellurite fiber in close proximately to the optical excitation. The microwave signals were delivered via a microwave generator (Agilent N5182A MXG) was used in combination with an amplifier (Minicircuits ZHL-16W-43-S+) to deliver approximately 1–4 W of RF power to the microwave antenna.

## Electronic supplementary material

Supplementary Information
