# Supplementary material for: Magnetically sensitive nanodiamond-doped tellurite glass fibers
Source: Sci Rep. 2018 Jan 19;8:1268. doi: 10.1038/s41598-018-19400-3 (PMC5775195; doi:10.1038/s41598-018-19400-3)
Supplement: Supplementary file 1 — Supplementary Information [file 41598_2018_19400_MOESM1_ESM.pdf]

Supplementary material

Magnetically sensitive nanodiamond-doped tellurite glass fibers

Yinlan Ruan[1,†], David A. Simpson[2,†], Jan Jeske[3], Heike Ebendorff-heidepriem[1], Desmond W. M. Lau[4], Hong Ji[1], Brett C. Johnson[5], Takeshi Ohshima[6], Shahraam Afshar V.[1,7], Lloyd Hollenberg,[2,5], Andrew D. Greentree[4], Tanya M. Monro[1,7], and Brant C. Gibson[4,*]

[1]ARC Centre of Excellence for Nanoscale BioPhotonics, Institute of Photonics and Advanced Sensing, The University of Adelaide, Adelaide, SA 5005, Australia.
[2]School of Physics, University of Melbourne, Parkville, VIC 3010, Australia.
[3]Chemical and Quantum Physics, School of Science, RMIT University, Melbourne VIC 3001, Australia.
[4]ARC Centre of Excellence for Nanoscale BioPhotonics, School of Science, RMIT University, Melbourne, VIC 3001, Australia.
[5]Centre for Quantum Computing and Communication Technology, School of Physics, University of Melbourne, Parkville, VIC 3010, Australia.
[6]National Institutes for Quantum and Radiological Science and Technology (QST), Takasaki, Gunma 370-1292, Japan.
[7]University of South Australia, Adelaide, SA 5000, Australia.
*corresponding author brant.gibson@rmit.edu.au
†These authors contributed equally to the work

Theoretical modelling of the ODMR spectra

By following the approach in Ref. [1], [2], each NV center was modelled as a seven-state system including the ground state spin triplet, the excited state triplet and a single state enabling non-spin-conserving, non-radiative transitions from the m_s=±1 excited states. Based on the transition rates given in Ref. [3], the emission rate was assumed to be Γ_1=(12ns)$^{-1}$, and the internal decay rates were assumed to be L_1=(24.9ns)$^{-1}$, L_2=(231ns)$^{-1}$, L_3=(462ns)$^{-1}$, respectively. The applied microwaves are modeled as driving coherent Rabi oscillations between the ground spin triplet states, assuming a Rabi frequency of Ω=0.9 MHz and an inhomogeneous dephasing rate of Γ_2^*=(0.01 μs)$^{-1}$ between the same states. The laser pumping rate is 1 MHz, and the NV inhomogeneous coherence time is 0.01 μs to account for the strong broadening observed in experiment. We then considered an ensemble of NV centers with four equally populated tetrahedral orientations, where one of the four orientations was aligned with the external magnetic field. We used the experimentally measured splitting of the ODMR to infer the magnetic field strength and used the same values in the modelling. The modelled results are shown in Fig. S1.

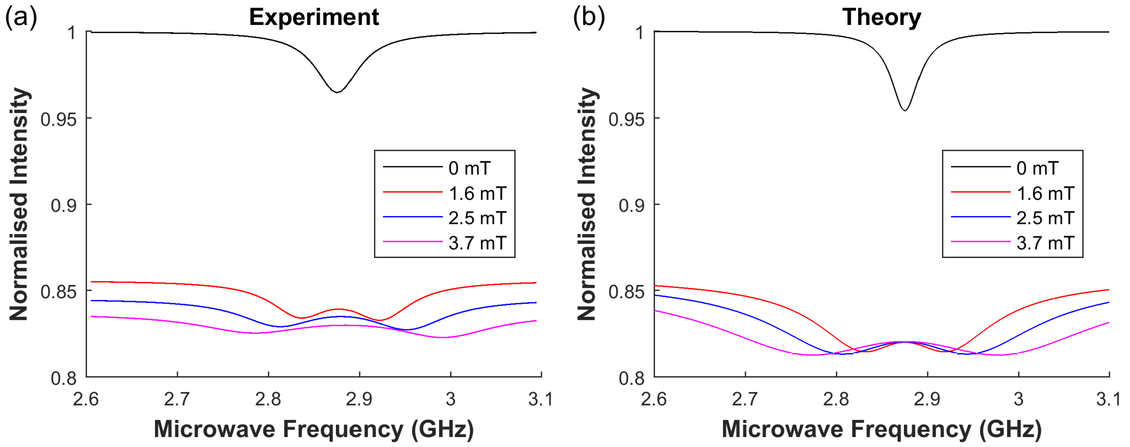

Fig. S1. Theoretical modeling of the ODMR spectra: (a) Experimentally fitted lines, (b) reproduction by theoretical modeling.

The experimental features are all reproduced qualitatively, including the reduction of fluorescence in the off-resonant region at 2.6 GHz and 3.1 GHz. We chose the parameters to reproduce these off-resonant features in particular, leaving small differences in the resonance width and contrast. The zero-field resonance for example is slightly thinner in the modeling than in the experiment, as wider peaks would obscure the off-resonant behavior for the non-zero magnetic field curves.

The precise value of the overall fluorescence reduction with the magnetic field is changed if there is constant background fluorescence present. Some background fluorescence is likely due to the residual fluorescence from the tellurite glass and constant fluorescence from NV centers, that were excited by the laser and collected through the fiber but which had minimal interaction with the RF driving fields. The observed reduction of the fluorescence is smaller with stronger background fluorescence. In Fig. S1 the observed values of normalized intensity for around 2.6 GHz and 3.1 GHz were well reproduced by the modeled curves by assuming a background fluorescence, which was 30% of the strongest signal (B = 0 mT). Our modeling shows that a complete removal of background fluorescence would move the range of the normalized intensities for the three curves to between 0.75 and 0.81, while an increase of the background fluorescence would shift the curves even closer to 1.

With these parameters we then modeled the reduction of off-resonant fluorescence as a function of magnetic field leading to figure 3(d) in the main manuscript.

References

[1] J. Jeske, J. H. Cole, and A. D. Greentree, "Laser threshold magnetometry" New J. Phys. **18** 013015 (2016).

[2] Jan Jeske, Desmond W. M. Lau, Xavier Vidal, Liam P. McGuinness, Philip Reineck, Brett C. Johnson, Marcus W. Doherty, Jeffrey C. McCallum, Shinobu Onoda, Fedor Jelezko, Takeshi Ohshima, Thomas Volz, Jared H. Cole, Brant C. Gibson, and Andrew D. Greentree, "Stimulated emission from nitrogen-vacancy centres in diamond," Nat. Commun.**8** 14000 (2017).

[3]. M. W. Doherty, N. B. Manson, P. Delaney, F. Jelezko, J. Wrachtrup, and L. C. Hollenberg, "The nitrogen-vacancy colour centre in diamond," Phys. Rep. **528**, 1-45 (2013).
